# Clustered Regularly Interspaced Short Palindromic Repeats (CRISPR) in Cardiovascular Disease: A Comprehensive Clinical Review on Dilated Cardiomyopathy

**DOI:** 10.7759/cureus.35774

**Published:** 2023-03-05

**Authors:** Vijaya Durga Pradeep Ganipineni, Sai Dheeraj Gutlapalli, Sumanth Danda, Sameer Krishna Prasad Garlapati, Daniel Fabian, Ikpechukwu Okorie, Jananthan Paramsothy

**Affiliations:** 1 Department of Internal Medicine, SRM Medical College Hospital and Research Centre, Chennai, IND; 2 Department of General Medicine, Andhra Medical College/King George Hospital, Visakhapatnam, IND; 3 Department of Internal Medicine, Richmond University Medical Center, Staten Island, USA; 4 Internal Medicine and Clinical Research, California Institute of Behavioral Neurosciences & Psychology, Fairfield, USA; 5 Department of Internal Medicine, Katuri Medical College & Hospital, Guntur, IND; 6 Department of Internal Medicine, Andhra Medical College/King George Hospital, Visakhapatnam, IND

**Keywords:** induced pluripotent stem cells, gene knockin, crispr/cas9 gene editing, genetic cardiomyopathy, cardiomyopathy, targeted therapeutics, dilated cardiomyopathy (dcm), gene editing, crispr/cas9, crispr

## Abstract

Dilated cardiomyopathy (DCM) is one of the most important causes of heart failure in developed and developing countries. Currently, most medical interventions in the treatment of DCM are mainly focused on mitigating the progression of the disease and controlling the symptoms. The vast majority of patients who survive till the late stages of the disease require cardiac transplantation; this is exactly why we need novel therapeutic interventions and hopefully treatments that can reverse the clinical cardiac deterioration in patients with DCM. Clustered regularly interspaced short palindromic repeats (CRISPR) technology is a novel therapeutic intervention with such capacity; it can help us edit the genome of patients with genetic etiology for DCM and potentially cure them permanently. This review provides an overview of studies investigating CRISPR-based gene editing in DCM, including the use of CRISPR in DCM disease models, phenotypic screening, and genotype-specific precision therapies. The review discusses the outcomes of these studies and highlights the potential benefits of CRISPR in developing novel genotype-agnostic therapeutic strategies for the genetic causes of DCM. The databases we used to extract relevant literature include PubMed, Google Scholar, and Cochrane Central. We used the Medical Subject Heading (MeSH) strategy for our literature search in PubMed and relevant search keywords for other databases. We screened all the relevant articles from inception till February 22, 2023. We retained 74 research articles after carefully reviewing each of them. We concluded that CRISPR gene editing has shown promise in developing precise and genotype-specific therapeutic strategies for DCM, but there are challenges and limitations, such as delivering CRISPR-Cas9 to human cardiomyocytes and the potential for unintended gene targeting. This study represents a turning point in our understanding of the mechanisms underlying DCM and paves the way for further investigation into the application of genomic editing for identifying novel therapeutic targets. This study can also act as a potential framework for novel therapeutic interventions in other genetic cardiovascular diseases.

## Introduction and background

Definition and background of dilated cardiomyopathy

Dilated cardiomyopathy (DCM) is a prevalent cause of heart failure worldwide [1]. It is defined by ventricular dilatation and systolic dysfunction of one or both ventricles [2]. It is a disease with high incidence and mortality rates [3]. DCM stands as a foremost etiology of heart failure, featuring a prevalence ranging from one in 250 to 500 individuals [1,4-7].

The disease is classed as idiopathic when all identifiable causes, with the exception of hereditary reasons, have been ruled out [2]. The median onset age for those affected ranges between 40 and 46 years [2]. The median period between diagnosis and transplant or death ranged from four to six years across all groups [2].

Overview of current treatment options and their limitations

The finding of a specific mutation does not govern treatment in patients with DCM [8]. Traditional therapeutic interventions, along with the promotion of a salubrious lifestyle, entail the administration of pharmacotherapies aimed at addressing heart failure with reduced ejection fraction, including angiotensin-converting enzyme (ACE) inhibitors, beta-adrenergic blockers, and aldosterone antagonists [8]. Some individuals with arrhythmias or conduction blocks and high arrhythmogenic mutations receive an implantable cardioverter defibrillator (ICD) or pacemaker [8]. Thus far, cardiac transplantation has stood as the sole curative option for the disease [9]. In fact, DCM is the most common diagnosis in patients undergoing cardiac transplantation [10]. As posited by Lipshultz et al. (2013), the primary goal of existing therapeutic modalities for DCM comprise the enhancement of survival rates, mitigation of disease progression, minimization of cardiovascular risk factors, and amelioration of associated symptoms [11]. In light of this, genetic therapies are picking up prominence in recent decades. They offer the potential to cure the disease without having to undergo cumbersome treatments and surgical transplant procedures and immunosuppression, which come with various complications.

CRISPR gene editing as a potential therapeutic approach

Despite the progress in understanding the genetic etiologies of DCM, the molecular mechanisms underlying the pathogenesis of DCM are not thoroughly understood. Therefore, current symptom-based therapeutic approaches do not address the underlying genetic basis of the disease, translating into a lack of preventive or disease-modifying therapies [12].

The diversity of gene-editing approaches is expanding due to the concerns and limitations of viral-mediated gene therapy, which continues to be essential for current gene therapy. A more appealing strategy is to correct the existing genetic abnormalities in situ, rather than introducing the therapeutic gene into a new and potentially problematic locus [13]. This method allows for repairing the pathological mutation while avoiding the risk of insertional oncogenesis. Nucleases, such as zinc finger nucleases (ZFN), transcription activator-like effector nucleases (TALENs), meganucleases, and most recently, the clustered regularly interspaced short palindromic repeats (CRISPR)/Cas system, have been developed for programmable gene editing [14-25]. While these tools can induce genome editing at targeted sites under controlled conditions, the CRISPR/Cas system has mostly replaced these earlier advances due to its relatively low cost, ease of use, and efficient and precise performance [24,25]. However, the CRISPR/Cas system is often delivered with adeno-associated virus (AAV) vectors, which do not completely eliminate the risks associated with viruses. Other delivery options are available to overcome this issue, each with its advantages and challenges. CRISPR/Cas9 is the most widely used tool for current genome editing among the CRISPR/Cas systems [26-33].

Objectives

With a specific focus on models for the investigation and therapy of DCM, this review seeks to provide an overview of the most current advancements in the field of genome editing using CRISPR/Cas9 and its applications in biomedical research.

Methodology

We conducted a review of the literature to identify studies related to the application of CRISPR in the context of DCM. Two reviewers (VG and DG) independently screened the titles and abstracts of articles retrieved from PubMed, Google Scholar, and Cochrane Central to identify potentially relevant studies. The following keywords were employed for the database search: dilated cardiomyopathy; cardiomyocytes; precision medicine; clinical-trail-in-a-dish; drug screening; phenotypic screens; atomic force microscopy; induced pluripotent stem cells; cardiovascular disease; human-induced pluripotent stem cell-derived cardiomyocytes; signal transduction; CRISPR; Duchenne muscular dystrophy; DMD; NHEJ; dystrophin; gene editing; CRISPR-associated protein 9; RNA splicing; cardiomyopathy; genetic therapy; muscular dystrophy; CRISPR-Cas systems; cardiomyopathy, dilated*/genetics; cardiomyopathy, dilated*/pathology; cell nucleas*/genetics; cells, cultured; gene expression profiling; gene knockout techniques; heart failure genetics; heart failure pathology; heart ventricles metabolism; heart ventricles pathology; myocardium metabolism; myocardium pathology; myofibroblasts pathology; RNA-Seq; single-cell analysis; and genetic transcription.

Full-text articles were then reviewed by the same reviewers to determine eligibility for inclusion. Any discrepancies were resolved through discussion and consensus. Data extraction was performed using Microsoft Excel (Microsoft Corporation, Redmond, WA) by the same reviewers (VG and DG). We included recently published articles as well as ongoing studies in our review. Details of the search strategy are available in the Appendix.

## Review

The role of genetic mutations in dilated cardiomyopathy

Overview of the Genetic Basis of Dilated Cardiomyopathy

Over 40 genes implicated in numerous cellular functions and structures have been identified as carrying pathogenic mutations [34]. Not all genetic variants identified in the patients are causative [35,36]. Studies have identified variants that are pathogenic or likely pathogenic in DCM patients [35,36]. Approximately 40% of cases of DCM are attributed to genetic causes [35,36], with the most common mutations occurring in genes responsible for cytoskeletal protein regulation, leading to sarcomere dysfunction, cellular metabolic pathways, and intracellular calcium homeostasis [3,37,38].

The pathogenesis of DCM involves defects in various cellular structures and processes. Hereditary DCM is responsible for 30-50% of cases, with an autosomal dominant inheritance pattern being the most frequent mode of transmission [39,40]. While less common, autosomal recessive, X-linked, and mitochondrial inheritance patterns have also been reported [34,41,42]. Molecular genetic testing for DCM involves testing a panel of genes, usually 30 to 50 through next-generation sequencing [34,42,43]. Mutations in genes encoding nuclear envelope proteins (such as lamin A and C), contractile apparatus (such as myosin heavy chain beta), membrane scaffolding (such as sarcoglycan), calcium handling proteins (such as phospholamban), and transcriptional and splicing machinery (such as ribonucleic acid-binding protein) have been identified as contributing factors [34,43]. Given the molecular complexity of DCM, it is likely that multiple factors contribute to contractile dysfunction, leading to cardiomyocyte death and myocardial fibrosis, which are hallmark features of DCM [34,43]. As such, genome editing systems hold great promise as a potential therapeutic avenue for DCM [3].

Genes Commonly Implicated in Dilated Cardiomyopathy

Table 1 shows the genes identified so far implicated in causing DCM.

**Table 1:** Genes and their corresponding proteins affected in dilated cardiomyopathy

Gene	Protein	Function
TNNT2	Cardiac troponin T	Sarcomere protein; muscle contraction
MYH6	Alpha-myosin heavy chain	Sarcomere protein; muscle contraction
MYH7	Beta-myosin heavy chain	Sarcomere protein; muscle contraction
MYBPC3	Myosin binding protein C	Sarcomere protein; muscle contraction
TPM1	Alpha-tropomyosin	Sarcomere protein; muscle contraction
ACTC1	Cardiac actin	Sarcomere protein, muscle contraction
TNNC1	Cardiac troponin C	Sarcomere protein; muscle contraction
TNNI3	Cardiac troponin I	Sarcomere protein; muscle contraction (recessive)
MYPN	Myopalladin	Sarcomere protein; Z-disc
VCL	Meta-vinculin	Sarcomere structure, Intercalated disc
ACTN2	Alpha-actinin 2	Sarcomere structure; anchor of myofibrillar actin
TTN	Titin	Sarcomere structure, extensible scaffold for other proteins
LMNA	Lamin A/C	Structure/stability of inner nuclear membrane; gene expression
SCN5A	Sodium channel	Controls sodium ion flux
RBM20	RNA binding protein 20	RNA binding protein of a spliceosome
ANKRD1	Ankyrin repeat domain, containing protein 1	Cardiac ankyrin repeat protein (CARP); localized to myopalladin/titin complex
LDB3	Cypher	Cytoskeletal assembly; targeting/clustering of membrane proteins
TCAP	Titin-cap or telethonin	Z disc protein that associates with titin; aids sarcomere assembly
P SEN 1/2	Presenilin 1/2	Transmembrane proteins, gamma-secretase activity
LAMA4	Laminin alpha 4	Extracellular matrix protein
CRYA B	Alpha B crystallin	Cytoskeletal protein
ILK	Integrin-linked kinase	Intracellular serine-threonine kinase; interacts with integrins
PLN	Phospholamban	Sarcoplasmic reticulum Ca2+ regulator; inhibits SERCA2 pump
ABCC9	SUR2A	Kir6.2 regulatory subunit, inwardly rectifying cardiac K- ATP channel
DES	Desmin	DAGC; transduces contractile forces
SGCD	Delta-sarcoglycan	DAGC; transduces contractile forces
NEBL	Nebulette	Binds actin; Z-disc assembly
NEXN	Nexilin	Cardiac Z-disc
CSRP3	Muscle LIM protein	Sarcomere stretch sensor/Z-disc
PDLIM3	LIM domain protein 3	Cytoskeletal protein

The most commonly affected genes include titin (TTN), which is the largest human gene and the most common cause of DCM [44-46]; LMNA, which is associated with a wide range of cardiac diseases and accounts for up to 10% of familial cases of DCM [46,47]; MYH7, which is associated with a broad spectrum of cardiac diseases and accounts for up to 8% of familial cases of DCM [46,48]; DSP, which is associated with arrhythmogenic right ventricular cardiomyopathy (ARVC) but can also cause DCM and accounts for up to 7% of familial cases; and RBM20, which is associated with DCM and accounts for up to 3% of familial cases [46,49,50].

It is important to note that these numbers may vary based on the population studied and other factors. Additionally, there are many other genes that have been implicated in DCM and the field of research on this topic continues to evolve [49].

Mechanisms Underlying the Development and Progression of the Disease

DCM is a complex and multifaceted disease that arises from several underlying genetic mechanisms [51]. One such mechanism is the dominant negative effect resulting from missense mutations that substitute a single amino acid [51]. The mutant protein expressed as a result interferes with the function of the normal allele, leading to disruptions in normal function [51]. Haploinsufficiency is another mechanism causing DCM, resulting from nonsense or frameshift mutations leading to the formation of a truncated or unstable protein [51]. This can lead to a deficiency of normal protein and the consequent loss of its function [51].

**Figure 1:**
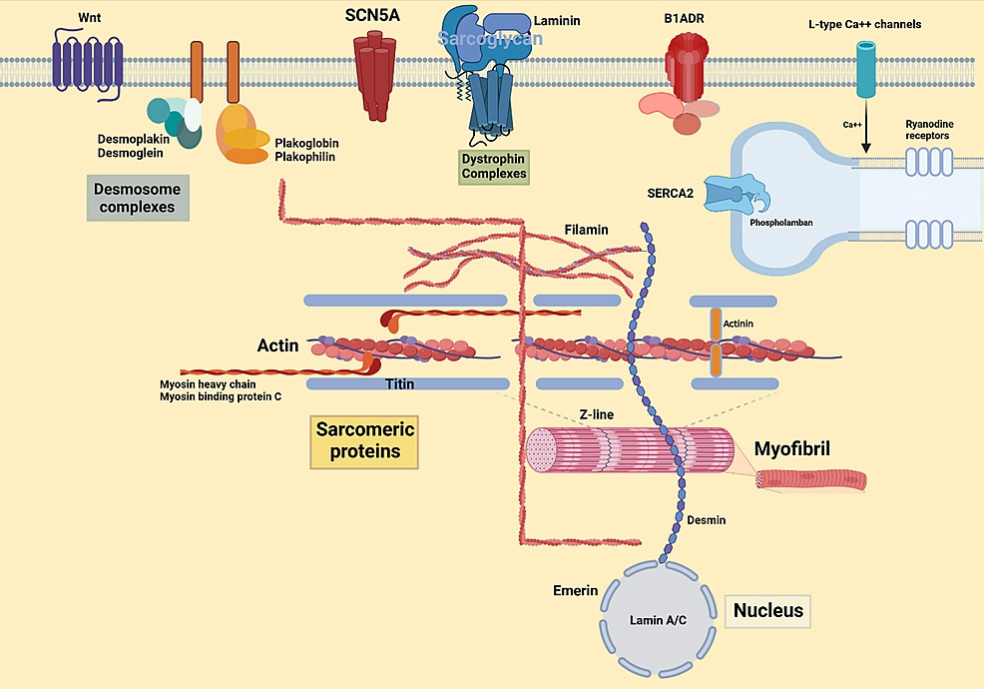
Picture of myocyte indicating multiple sites of abnormal gene products associated with cardiomyopathy Image credits: Ganipineni V

Deletion of the entire genome/exon is a rare cause of cardiomyopathies except for dystrophinopathies [51]. Furthermore, locus heterogeneity is another important mechanism where multiple genes are implicated in the phenotypic expression of DCM [51]. Finally, allelic heterogeneity, a phenomenon where different mutations in a single gene produce the same phenotype of DCM, is another mechanism driving the disease. These mechanisms disrupt the expression and function of myriad proteins [51]. These can be broadly divided into sarcomeric proteins, Z-disc/cytoskeleton, sarcolemmal membrane, intercalated discs/desmosomal complex, and nuclear membrane [51]. Figure 1 shows the intracellular structure of a myocyte indicating multiple sites of abnormal gene products associated with cardiomyopathy.

Mutations in the sarcolemmal membrane protein SCN5A are less commonly associated with DCM and more commonly associated with Brugada syndrome [49,51]. Alterations in the dystrophin protein, which is present in both skeletal and cardiac muscle, cause Duchenne muscular dystrophy (DMD) and can lead to DCM by the age of 20, resulting in death [49,51]. Defects in nuclear membrane proteins, lamin A/C and emerin, have a distinct presentation with a high prevalence of atrial arrhythmias and conduction defects [49,51]. Intercalated discs/desmosomal complex mutations cause loss of intercellular connections and cell death, which is replaced by fibrofatty tissue that is highly arrhythmogenic, resulting in ARVC [49,51].

A thorough understanding of the genes and their proteins affected in DCM is crucial in the development of effective treatment strategies. Identifying the underlying genetic mechanisms can provide insight into the pathophysiology of DCM and aid in the development of targeted therapies that may ultimately improve outcomes for patients with this debilitating disease.

Overview of CRISPR gene editing technology

CRISPR-Cas9 System and How it Works

**Figure 2:**
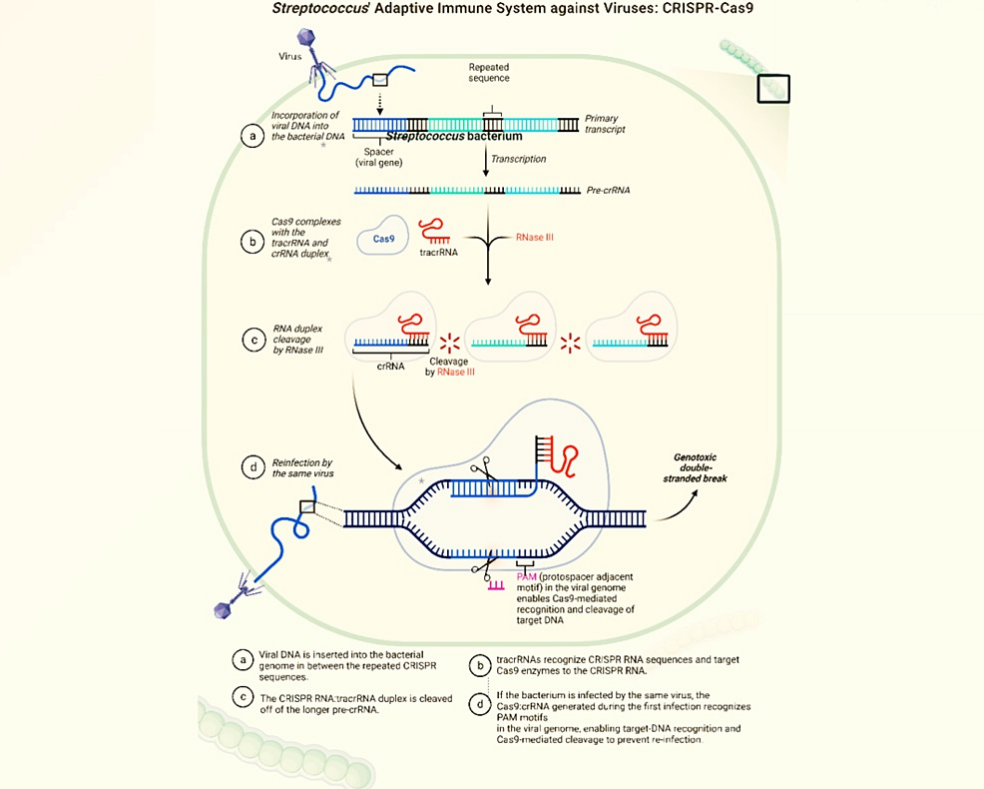
Function of CRISPR-Cas9 in the bacterium CRISPR: clustered regularly interspaced short palindromic repeats; crRNA: CRISPR RNA; PAM: protospacer adjacent motif. Image Credits: Ganipineni V

CRISPR/Cas9 is a gene-editing tool that is derived from the bacterial immune system [3]. This technology employs a small RNA molecule as a guide to direct an endonuclease (Cas9) to a precise location on the DNA [3,52]. The CRISPR/Cas9 methodology relies on the precise cutting of DNA, followed by natural repair processes [3]. The technique can correct the expression levels of dysfunctional genes and promote the loss or gain of function [3,53]. Initially, the CRISPR genes are transcribed into a single-stranded RNA that undergoes processing to generate a small CRISPR RNA (crRNA) [3,53]. The crRNA then guides the nucleolytic activity of the Cas9 enzyme to degrade specific nucleic acids [3,52]. The Cas9 endonuclease, derived from *Streptococcus pyogenes*, has been extensively utilized in the genomic editing of various species and cell types [3]. Figure 2 demonstrates the mechanism of CRISPR in the bacterium *Staphylococcus aureus*.

Overview of the CRISPR-Based Gene Editing Techniques

In the CRISPR/Cas9 system, the RNA sequences are programmed to target specific genes, and the Cas9 enzyme cuts the DNA at the targeted site [13]. This creates a double-strand break that can be repaired by one of two mechanisms: non-homologous end joining (NHEJ) or homology-directed repair (HDR) [54]. Due to the error-prone nature of NHEJ, the cut site frequently experiences insertions or deletions (indels), which result in the knockout of the target gene [54]. HDR, on the other hand, can be used to introduce specific changes in the DNA sequence, such as knock-in mutations [54].

More recently, CRISPR has been adapted to allow for base editing (BE) and prime editing (PE) [13,55,56]. BE uses a fusion protein of the Cas9 enzyme and a cytidine deaminase to convert a C-G base pair to a T-A pair or an A-T pair to a G-C pair, without creating double-strand breaks [13,55]. This method has been used to create specific point mutations in human cells with high efficiency and specificity, as reported in a study by Gaudelli et al. (2017) [55].

PE, a newer CRISPR-based technique, was developed to address some of the limitations of BE and traditional CRISPR [13,56]. PE uses a fusion protein of the Cas9 enzyme and reverse transcriptase to make precise edits at specific locations in the genome, without creating double-strand breaks [13,56]. This technique has been used to introduce specific mutations and insertions, as reported in a study by Anzalone et al. (2019) [13,56].

**Figure 3:**
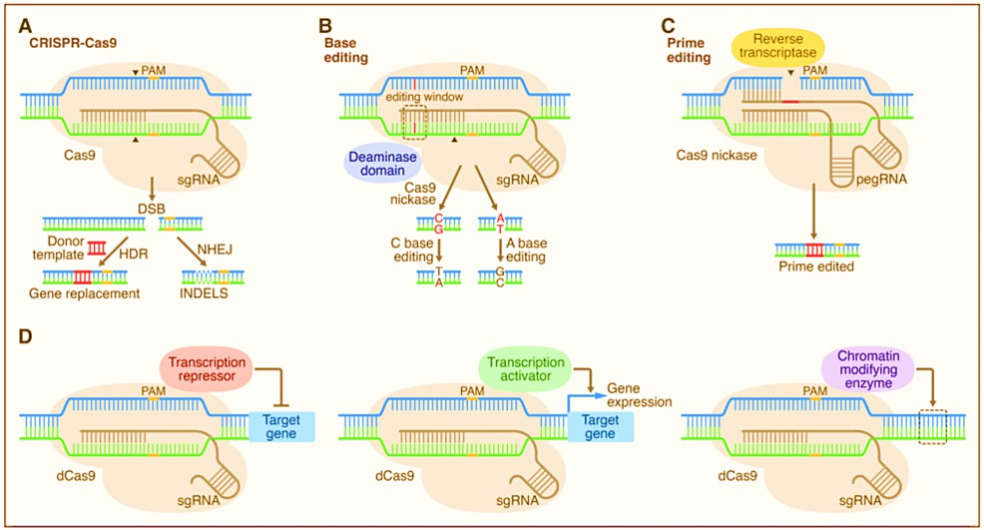
Gene editing using CRISPR-Cas9 A. The CRISPR/Cas9 system relies on the targeting of the Cas9 nuclease to a specific DNA sequence via a complementary guide RNA (gRNA) molecule [3]. Once the Cas9-gRNA complex has located the target site, the Cas9 enzyme creates a double-stranded break (DSB) in the DNA [3]. Subsequently, the DNA repair machinery is triggered, which can result in two major repair pathways: non-homologous end joining (NHEJ) or homology-directed repair (HDR) [3]. In the NHEJ pathway, the DNA strands are rejoined by enzymes, but this process can result in errors, such as deletions or insertions, which can alter the genetic code of the cell. In the HDR pathway, a template DNA strand is used to repair the broken strand, leading to a more precise repair [3]. B. Base editing: The CRISPR/Cas9 system is used to target a specific genomic location. An engineered base editor complex, consisting of a catalytic domain and a guide RNA, is then delivered to the target site [13,55]. The catalytic domain contains an enzyme that can directly convert one nucleotide to another without breaking the DNA strand. In this example, a cytosine (C) is converted to a thymine (T) and guanine (G) to adenine (A) by the base editor [13,55]. C. Prime editing: The CRISPR/Cas9 system is used to nick one of the DNA strands at a specific genomic location [13,56]. A prime editing complex, consisting of a reverse transcriptase, a nickase Cas9, and a prime editing guide RNA (pegRNA), is then delivered to the target site [13,56]. The pegRNA contains a template sequence for the desired edit, which is reverse transcribed into the nicked DNA strand [13,56]. The nick is then repaired using the template, resulting in the desired edit without inducing double-strand breaks [13,56]. CRISPR: clustered regularly interspaced short palindromic repeats; PAM: protospacer adjacent motif. Image Credits: Ganipineni V

Figure 3 shows the mechanism of gene editing by CRISPR-Cas9 and its recent advances in BE and PE.

The CRISPR system has evolved to include a variety of gene-editing techniques, including knockout, knock-in, BE, and PE [13]. Each of these techniques has unique advantages and limitations, and the choice of technique will depend on the specific research question and application [13]. The continued development of CRISPR-based gene editing techniques holds great promise for the treatment of genetic diseases and for the advancement of basic science.

Advantages of Using CRISPR for Gene Editing

The CRISPR/Cas9 system represents an economical and straightforward approach relative to extant genome editing strategies, including plasmid vectors and restriction enzymes [57]. Cas9, guided by RNA, exhibits exceptional specificity, efficiency, and ease of design, rendering it highly adaptable for multiplexed gene editing applications in a multitude of cellular systems and organisms [57]. It offers a high level of customization, as the retargeting of Cas9 to new DNA sequences only requires the purchase of a pair of oligos encoding the 20-nt guide sequence [22,23,57]. In contrast, constructing new pairs of TALENs for a new DNA sequence requires a substantially greater amount of hands-on time [22,23,55,58-60]. The Cas9 system has a unique cleavage pattern that results in a blunt cut between the 17th and 18th bases of the target sequence, located 3 bp 5' of the protospacer adjacent motif [30,32]. Furthermore, mutations can be introduced in the RuvC or HNH nuclease domains of the Cas9 enzyme to convert it into a DNA-nicking enzyme [30,32]. In contrast, TALENs create non-specific cuts in the 12-24-bp linker region between the two TALEN monomer-binding sites [61]. Both Cas9 and TALENs have been demonstrated to effectively induce genome editing across various organisms and cell types [22,23,55,58-60]. However, the Cas9 system has the added advantage of targeting multiple genomic loci at once by co-delivering a combination of single guide RNAs (sgRNAs) to the cells [41]. This property greatly enhances the flexibility and versatility of the Cas9 system and makes it an attractive tool for various applications in engineering various genes. While CRISPR-Cas9 has shown promise in treating some forms of DCM, it also has several limitations that must be addressed.

Studies investigating the use of CRISPR in dilated cardiomyopathy

Overview of Studies Investigating CRISPR-Based Gene Editing in Dilated Cardiomyopathy

Table 2 provides a summary of these studies.

**Table 2:** Studies investigating CRISPR-based gene editing in DCM Targeting the genes of interest to study the mechanistic underpinnings of disease and develop a hypothesis. CRISPR: clustered regularly interspaced short palindromic repeats; DCM: dilated cardiomyopathy; iPSC: induced pluripotent stem cell; CMs: cardiomyocytes.

Study ID	Gene	Delivery vehicle		Type of delivery	Model			Type of iPSC	
					Organism	iPSC	Non-human primates	Mutant iPSC	Patient-derived iPSC
Carroll et al. (2016) [62]	Myh6		AAV9-sgRNA/intraperitoneal injection		Cardiac-specific Cas9 transgenic mice				
Rebs et al. (2020) [63]	RBM 20 - heterozygous missense mutation c.C1900T (p.R634W), located in the RS domain of RBM20					Human iPSC			Patient-derived: -R634W-iPSC-CMs Isogenic Control: - Rescue DCM-R634W-iPSCs
Hakim et al. (2018) [64]	Nonsense mutation in exon 23 of the dystrophin gene		AAV-sgRNA IV injection via tail vein	Dual vector approach	6-week-old mice transfection				
Barndt et al. (2021) [65]	PLN (phospholamban)-R9C mutation		Plasmid transfection			Human iPSC		Mutant iPSC Isogenic control iPSC	
Sun et al. (2020) [66]	CSRP3 gene (MLP) - compound heterozygous 13 bp deletion/1 bp insertion		Selected sgRNA (TGGGGCGGAGGCGCAAAATG) was cloned into the epiCRISPR plasmid containing Cas9 and 2A-Puro	epiCRISPR plasmid was introduced into the H9 using a 4D nucleofector system		Human iPSC		Human mutant iPSC	
Sui et al. (2018) [67]	Dystrophin - exon 51 knock out			Cytoplasm microinjection of Cas9 mRNA and single guide RNA (sgRNA)	Co-injection of Cas9 mRNA and sgRNA targeting exon 51 into rabbit zygotes				
El Refaey et al. (2017) [68]	Dystrophin - exon 23-point mutation knockout		Adeno-associated virus vector - serotype rh.74	AAV delivered to mdx/Utr+/− mice systemically via a retro-orbital approach	Mice neonate transfection				
Dave et al. (2022) [69]	PLN9 (phospholamban) - deletion of Arg14 (R14del)		Cardiotropic adeno-associated virus-9 (AAV9)						
Xu et al. (2021) [70]	Troponin T (TnT), TnT-R141W		Puro plasmid			Human iPSC		Human mutant iPSC	

DCM Disease Model Using CRISPR

**Figure 4:**
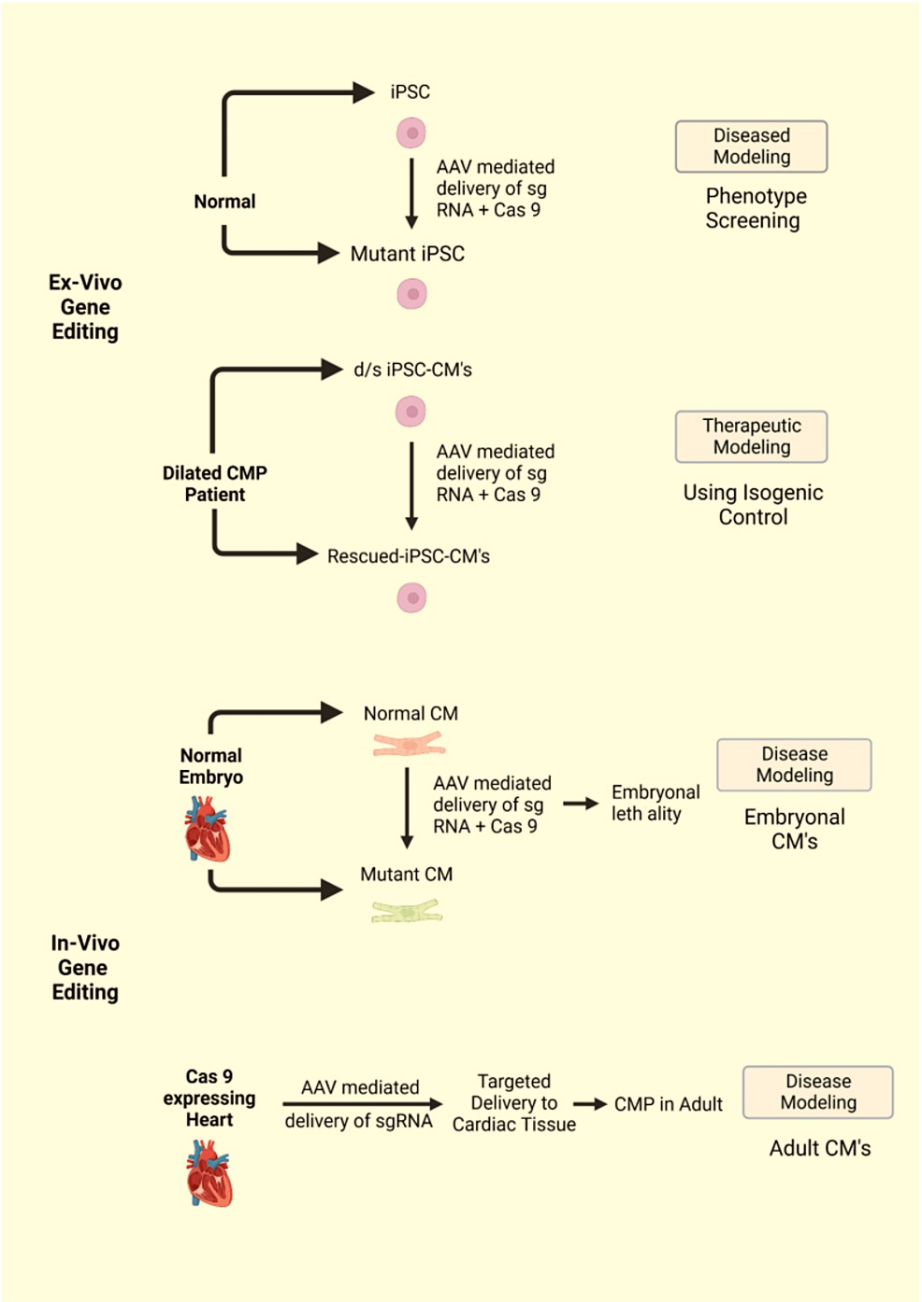
Applications of CRISPR in dilated cardiomyopathy 1. Ex-vivo gene editing: The figure illustrates ex-vivo gene editing techniques for disease modeling and therapeutic modeling. (A) shows gene knockout for disease modeling, where AAV-mediated delivery of guide RNA along with Cas9 is used to convert induced pluripotent stem cells (iPSCs) into mutant iPSCs. (B) shows gene knock-in for therapeutic modeling, where AAV-mediated delivery of guide RNA along with Cas9 is used to convert diseased patient iPSCs into normal iPSCs. 2. In-vivo gene editing: The figure illustrates in-vivo gene editing techniques for disease modeling. (A) AAV-mediated delivery of guide RNA along with Cas9 is done to the zygote of a normal mouse. This leads to the introduction of a gene mutation in the embryo, which ultimately leads to its death. This approach is useful for disease modeling studies in vivo. (B) AAV-mediated delivery of modified Cas9 is done to an adult mouse to create a Cas9-expressing heart. This is followed by transfection of the mouse with AAV-mediated delivery of guided RNA. This approach limits the action of Cas9 to the targeted tissue, such as the myocardium, and helps avoid off-target effects. This method is useful for disease modeling studies in adult animals. CRISPR: clustered regularly interspaced short palindromic repeats; iPSC: induced pluripotent stem cell; CM: cardiomyocyte; AAV: adeno-associated virus. Image Credits: Ganipineni V

CRISPR-Cas9 is a very useful tool when it comes to studying the actions of various genes to better understand the pathophysiological process that happens in the genetic diseases of the cardiovascular system [62]. DCM disease models were developed in the lab using gene editing to recapitulate the disease process in vitro [9]. Figure 4 illustrates the application of CRISPR for modeling DCM.

CRISPR in Phenotypic Screening

Phenotypic screening is a method used in drug discovery and development to identify potential therapeutic compounds that affect the observable characteristics, or "phenotypes", of cells or organisms [9]. There is progress in understanding the genetic etiologies but not the underlying pathogenesis of DCM causing a lack of disease-specific therapies [9]. Induced pluripotent stem cell-derived cardiomyocytes (iPSC-CMs) allow us to recapitulate the disease phenotypes in vitro [9]. Phenotypic screening on these cells would allow us to test different drug treatments to see if the drugs rescued the contractile and metabolic function in the in vitro DCM model [9]. In a recent study by Gil et al., the authors used CRISPR gene editing to correct the patient’s iPSC-CMs to use them as an isogenic control [9]. Surprisingly, after correcting the mutation with CRISPR, the cardiomyocytes exhibited contractility levels comparable to iPSC-CMs obtained from healthy donors [9]. CRISPR can be utilized in this way for this novel genotype-agnostic therapeutic strategy for the genetic causes of DCM [9].

Several studies have employed CRISPR gene editing technology to create disease models and explore potential gene therapies in the context of cardiac disease and dysfunction. Table 2 can be referred to for a summary of these studies. Carroll et al. (2016) utilized a cardiac-specific Cas9 transgenic mouse model to demonstrate the efficacy of CRISPR/Cas9-based editing in adult cardiomyocytes [62]. The study employed a proof-of-concept approach to editing the Myh6 gene, resulting in cardiomyopathy and heart failure in the cardiac-specific Cas9 mouse [62]. Additionally, the study overcame limitations associated with traditional CRISPR/Cas9, such as off-target effects and embryonic lethality [62]. The use of tissue-specific editing by CRISPR/Cas9 could also provide an advantage for studying the effects of genes in specific tissues [62].

In another study, Barndt et al. (2021) generated PLN-R9C induced pluripotent stem cells (iPSCs), a new disease model using CRISPR/Cas9 single nucleotide editing technology, for studying the molecular mechanisms underlying phospholamban (PLN) mutation-related DCM [65]. The genome-edited iPSC line showed typical pluripotent cell morphology, robust expression of pluripotency markers, normal karyotype, and the capacity to differentiate into all three germ layers in vitro [65]. The generation of isogenic iPSC DCM models provides a valuable resource for studying the pathological mechanisms of DCM caused by the PLN-R9C mutation [65].

Furthermore, Sun et al. (2020) investigated the role of the CSRP3 gene in cardiac muscle physiology and pathology [66]. The researchers generated CSRP3 homozygous knockout embryonic stem cells using the CRISPR/Cas9 system [66]. The study provides a novel resource to understand functions mediated by MLP during human cardiomyopathy and heart failure [66].

In yet another study, Sui et al. (2018) generated a rabbit model of DMD using CRISPR/Cas9 technology [67]. The researchers designed a pair of sgRNAs targeting exon 51 of the DMD gene, which is commonly mutated in human DMD patients [67]. The resulting knockout DMD rabbits showed typical DMD phenotypes, including elevated serum creatine kinase levels, severely impaired physical activity, and progressive muscle necrosis and fibrosis [67]. This novel rabbit DMD model created with the CRISPR/Cas9 system mimics the functional defects and histopathological changes in DMD patients and could be a valuable resource for preclinical studies [67].

DCM Therapeutic Model Using CRISPR/Cas9

Dave et al. (2022) aimed to assess the therapeutic effects of CRISPR/Cas9-mediated genome editing on cardiac function in mice carrying a mutation in the PLN9 gene [69]. The authors employed a cardiotropic AAV9 vector to deliver CRISPR/Cas9 and guide RNA (gRNA) effectively disrupting the gene and evaluated the efficacy of the gene editing using droplet digital polymerase chain reaction and next-generation sequencing (NGS)-based amplicon sequencing [69]. The results indicated that mutant mice had bi-ventricular dilation and increased stroke volume compared to wild-type mice, with a higher propensity for sustained ventricular tachycardia [69]. This is followed by in vivo gene editing to correct the induced mutation, which significantly reduced end-diastolic and stroke volumes and susceptibility to ventricular tachycardia [69]. The findings suggest that CRISPR/Cas9-mediated gene editing may provide a potential therapeutic option for cardiac disorders associated with the PLN9 gene mutation [69].

Similarly, the study conducted by Hakim et al. (2018) aimed to examine dystrophin expression and disease rescue in mice with a nonsense mutation in exon 23 of the dystrophin gene using a dual-vector AAV-CRISPR approach [64]. The authors found that increasing the dose of the gRNA vector significantly enhanced dystrophin expression in the heart and resulted in body-wide dystrophin restoration in skeletal muscle [64]. These results suggest that increasing the gRNA vector dose is necessary to achieve sustained skeletal muscle and heart rescue in DMD [64].

Finally, El Refaey et al. (2017) utilized an AAV vector of serotype rh.74 to deliver CRISPR/Cas9 systemically to mdx mice via a retro-orbital approach causing mice neonate transfection [68]. The study aimed to knockout exon 23 of the dystrophin gene [68]. The results demonstrated the feasibility of systemic AAV rh.74-mediated CRISPR/Cas9 delivery for targeted genome editing in mdx mice [68].

Taken together, these studies provide compelling evidence for the potential use of CRISPR/Cas9-mediated gene editing in cardiac disorders. The findings provide a valuable contribution to the development of diagnostic methods and therapeutic targets using patient-specific iPSC models and hold promise for future therapeutic applications in cardiac diseases. Although, further studies are required to validate the efficacy and safety of CRISPR/Cas9-mediated gene editing in clinical settings.

Genotype-Specific Precision Therapies

An integrated platform was created by Xu et al. (2021) to enhance the understanding of molecular functions related to cardiomyopathies, particularly DCM [70]. The platform utilizes iPSC-CMs that carry disease-causing mutations to establish an isogenic system for testing the analysis of molecular disease phenotypes related to cardiomyopathies, including dysregulated contractility, reduced beating force, and abnormal calcium handling [70]. The researchers utilized a non-negative blind deconvolution-based convergent peak fitting approach to evaluate the data, which facilitated automated curve fitting for calcium transients, contraction motion recordings, and contractile force responses in the iPSC-CM models [70]. The platform aims to design more accurate diagnostic tools for cardiomyopathies, particularly DCM, which is a common cause of heart failure [70]. By providing a more comprehensive and integrated analysis of crucial molecular parameters such as contractility, beating force, and calcium transients in iPSC-CMs, the platform may help identify disease-specific alterations that are suitable for therapeutic targeting using small molecules [70]. Furthermore, the use of iPSC and CRISPR/Cas-edited models can address personalized drug responses and intraindividual variability in patient populations, which can limit conclusiveness in human patient-specific models [70].

Although gene disruption using CRISPR/Cas9 is not always effective in treating genetic cardiomyopathies due to haploinsufficiency, precise gene correction strategies using base editors and prime editors have been applied and are potentially well-suited for correcting RBM20 mutations that cause cardiomyopathy due to a mix of loss-of-function and pathogen gain-of-function [71]. A recent study by Nishiyama et al. (2022) demonstrated precise gene editing of RBM20 mutations by BE and PE, which reduced toxic ribonucleoprotein (RNP) granules present in cardiomyocytes and rescued cardiac dysfunction in mice [71]. However, DNA off-target editing is a potential problem for clinical translation, and although ABE8e can efficiently edit the R634Q mutation, it can induce bystander mutations due to its wider editing window [71]. To reduce off-target effects, ABEmax and PE3b with a sgRNA recognizing the edited sequence can be considered [71]. AAV is the most commonly used viral vector for gene delivery to the heart, but the large size of the BE and PE systems pose challenges to efficient delivery [72]. To mitigate the incidence of off-target cleavage, Naeem and colleagues (2020) proposed a novel double-nicking strategy utilizing a Cas9 nickase mutant paired with guide RNAs [73]. The nickase mutant is a modified form of the spCas9 enzyme, which is able to produce more precise and specific gene editions, thereby reducing off-target effects [66].

Identification of Limitations and Potential Concerns of These Studies

Traditional gene therapy utilizing viral vector delivery of therapeutic transgenes has been associated with insertional oncogenesis and immunogenic toxicity [3]. However, the advent of CRISPR technology has allowed for in vivo or ex vivo therapeutic gene editing, depending on the tissue of interest [3]. For blood disorders such as β-thalassemia and sickle cell disease, infusion of ex vivo edited patient-derived hematopoietic stem cells has shown promise in treating those diseases [74]. Conversely, efficient and safe delivery systems are required for genome editing in cardiomyopathies. AAV has emerged as a leading candidate for delivering CRISPR/Cas9 components to the heart [74]. However, the limited packaging capacity of AAV (≈4.7 kb) necessitates separate vectors for the most commonly used Cas9 from *Streptococcus pyogenes* and its single-guide RNA [74]. To address this issue, several small Cas9 orthologs have been engineered [74]. The delivery of base editors (BEs) or prime editors (PEs) is also limited by AAV packaging capacity, although a dual-AAV system utilizing trans-splicing inteins has been shown to reconstitute full-length BEs and PEs [74].

Gene disruption can be deployed to target dominant-negative or pathogenic gain-of-function mutations by preventing the expression of the mutant allele and eliminating the dysfunctional protein [24,27-33]. However, many genetic mutations cannot be corrected by these strategies because of the haploinsufficiency of the modified protein product [24,27-33]. Precise gene correction strategies allow the potential correction of monogenic mutations responsible for various cardiomyopathies by gene editing [24,27-33]. Therapeutic approaches using BE and PE have been applied for DMD, progeria, inheritable liver, and eye disorders in vivo [24,27-33].

Challenges and limitations of using CRISPR in dilated cardiomyopathy

One of the primary limitations of using CRISPR-Cas9 for treating DCM is the delivery of CRISPR-Cas9. While the technique has been successfully used in animal studies, delivering CRISPR-Cas9 to human cardiomyocytes remains challenging. Currently, the most common method for delivering CRISPR-Cas9 is through viral vectors, which have the potential to cause immune reactions and other side effects [13].

Another limitation is the potential for off-target effects. While CRISPR-Cas9 is highly specific, it can sometimes target unintended regions of the genome, leading to unintended effects [71]. This is a particular concern in the context of DCM, where the genes involved are often complex and have multiple functions. Additionally, CRISPR-Cas9 is limited by the fact that it can only correct mutations that are located in certain regions of the genome, such as exons. Some mutations that cause DCM may be located in other regions of the genome, such as introns, and thus cannot be corrected using CRISPR-Cas9.

Finally, another challenge of using CRISPR-Cas9 to treat DCM is that the disease is often caused by mutations in multiple genes, making it difficult to target all the relevant genes using a single CRISPR-Cas9 treatment. In some cases, a combination of different treatments may be necessary to effectively treat DCM.

Future directions and outlook for CRISPR gene editing in dilated cardiomyopathy

Drawing inspiration from a bacterial defense mechanism against phages, the CRISPR/Cas9 methodology has emerged as a dynamic and versatile platform for editing DNA, successfully surmounting numerous obstacles encountered by conventional techniques that are both labor-intensive and time-consuming, gaining significant prominence as the preferred modality for both disease modeling and therapeutic applications [70].

Future research and development are required to overcome the current limitations of CRISPR gene editing in DCM. One limitation is redundancy, which can be overcome by using more precise and targeted nucleases like saCas9 and Cas9 nickase [66]. Another limitation is mosaicism, which can be addressed by using cardiac-specific genome editing with CRISPR/Cas9. This can be achieved by modifying the construct expressing Cas9 from *Streptococcus pyogenes* together with a green fluorescent protein (GFP) tag and replacing the CBh promoter with the promoter for a heart-specific gene of interest, thereby allowing the expression of Cas9 exclusively in cardiomyocytes [67]. Furthermore, certain genes exhibit enhanced susceptibility to genetic manipulation [75], necessitating disease modeling and phenotype screening to elucidate underlying mechanistic processes and comprehend the roles of different genes. Another limitation is with packaging, which can be overcome by utilizing a dual-vector approach in which the SpCas9 and the sgRNAs are delivered to the heart by separate AAV constructs [68]. This allows for easy modification of the ratio of SpCas9 and sgRNA, which may be critical for efficient targeting [68]. Finally, the depletion of the sgRNA-carrying vector can be overcome by increasing the dose of the gRNA vector dose [69].

Given that the heart is highly vascularized, other delivery strategies, such as nanoparticle-mediated delivery, might overcome the bottlenecks of AAV delivery if they could be delivered efficiently [74].

Further research into these methods and potential novel approaches may help to overcome the current limitations of CRISPR gene editing in DCM.

## Conclusions

The potential of CRISPR technology in the cardiac field remains largely untapped, as evidenced by the meager number of published studies. While prior investigations have chiefly focused on the genetic editing of cardiomyocytes and human induced pluripotent stem cells, these endeavors have not comprehensively explored the vast possibilities of this cutting-edge technology. Given its versatility, the continued progression and popularization of CRISPR are imminent, making it an indispensable tool for an array of applications. Our narrative review adds to the burgeoning body of literature in this area and provides a conceptual foundation for elucidating the intricate interplay at work.

In conclusion, CRISPR gene editing has demonstrated remarkable potential for developing precise and genotype-specific therapeutic strategies for DCM. By using CRISPR to create disease models and conduct phenotypic screening, researchers can study the actions of various genes and better understand the pathophysiological processes that occur in genetic diseases of the cardiovascular system. Despite the significant progress made thus far, the clinical application of CRISPR-Cas9 for treating DCM is not without challenges and limitations. One of the primary limitations is the delivery of CRISPR-Cas9 to human cardiomyocytes, which remains challenging due to concerns about immune reactions and other side effects. Additionally, while CRISPR-Cas9 is highly specific, it can sometimes target unintended genes. Nevertheless, advances in delivery methods and continued research into the safety and efficacy of CRISPR-based gene therapies hold great promise for developing new treatments for DCM and other genetic disorders. Moreover, to fully unlock the potential of CRISPR in the realm of cardiovascular pathology, research efforts should extend to the examination of large animal models, specifically non-human primates, whose cardiac anatomy and physiologic profiles parallel those of humans. This strategy will afford valuable insight into the vast potential of CRISPR for a range of therapeutic goals.

## Appendices

Search strategy

PubMed Central

((("precision medicine"[MeSH Terms] OR ("precision"[All Fields] AND "medicine"[All Fields]) OR "precision medicine"[All Fields] OR ("induced pluripotent stem cells"[MeSH Terms] OR ("induced"[All Fields] AND "pluripotent"[All Fields] AND "stem"[All Fields] AND "cells"[All Fields]) OR "induced pluripotent stem cells"[All Fields]) OR (("phenotype"[MeSH Terms] OR "phenotype"[All Fields] OR "phenotypes"[All Fields] OR "phenotyped"[All Fields] OR "phenotypic"[All Fields] OR "phenotypical"[All Fields] OR "phenotypically"[All Fields] OR "phenotyping"[All Fields] OR "phenotypings"[All Fields]) AND ("diagnosis"[MeSH Subheading] OR "diagnosis"[All Fields] OR "screening"[All Fields] OR "mass screening"[MeSH Terms] OR ("mass"[All Fields] AND "screening"[All Fields]) OR "mass screening"[All Fields] OR "early detection of cancer"[MeSH Terms] OR ("early"[All Fields] AND "detection"[All Fields] AND "cancer"[All Fields]) OR "early detection of cancer"[All Fields] OR "screen"[All Fields] OR "screenings"[All Fields] OR "screened"[All Fields] OR "screens"[All Fields])) OR "Clinical-trial-in-a-dish"[All Fields] OR ("human-induced"[All Fields] AND ("pluripotence"[All Fields] OR "pluripotency"[All Fields] OR "pluripotent"[All Fields]) AND ("plant stems"[MeSH Terms] OR ("plant"[All Fields] AND "stems"[All Fields]) OR "plant stems"[All Fields] OR "stem"[All Fields] OR "microscopy, electron, scanning transmission"[MeSH Terms] OR ("microscopy"[All Fields] AND "electron"[All Fields] AND "scanning"[All Fields] AND "transmission"[All Fields]) OR "scanning transmission electron microscopy"[All Fields]) AND "cell-derived"[All Fields] AND ("cardiomyocyte s"[All Fields] OR "cardiomyocytic"[All Fields] OR "myocytes, cardiac"[MeSH Terms] OR ("myocytes"[All Fields] AND "cardiac"[All Fields]) OR "cardiac myocytes"[All Fields] OR "cardiomyocyte"[All Fields] OR "cardiomyocytes"[All Fields])) OR ("drug evaluation, preclinical"[MeSH Terms] OR ("drug"[All Fields] AND "evaluation"[All Fields] AND "preclinical"[All Fields]) OR "preclinical drug evaluation"[All Fields] OR ("drug"[All Fields] AND "screening"[All Fields]) OR "drug screening"[All Fields]) OR ("clustered regularly interspaced short palindromic repeats"[MeSH Terms] OR ("clustered"[All Fields] AND "regularly"[All Fields] AND "interspaced"[All Fields] AND "short"[All Fields] AND "palindromic"[All Fields] AND "repeats"[All Fields]) OR "clustered regularly interspaced short palindromic repeats"[All Fields] OR "crispr"[All Fields] OR "crisprs"[All Fields]) OR ("gene editing"[MeSH Terms] OR ("gene"[All Fields] AND "editing"[All Fields]) OR "gene editing"[All Fields]) OR ("crispr associated protein 9"[MeSH Terms] OR "crispr associated protein 9"[All Fields] OR "crispr associated protein 9"[All Fields]) OR ("rna splicing"[MeSH Terms] OR ("rna"[All Fields] AND "splicing"[All Fields]) OR "rna splicing"[All Fields]) OR ("gene editing"[MeSH Terms] OR ("gene"[All Fields] AND "editing"[All Fields]) OR "gene editing"[All Fields]) OR ("genetic therapy"[MeSH Terms] OR ("genetic"[All Fields] AND "therapy"[All Fields]) OR "genetic therapy"[All Fields]) OR ("crispr cas systems"[MeSH Terms] OR ("crispr cas"[All Fields] AND "systems"[All Fields]) OR "crispr cas systems"[All Fields] OR ("crispr"[All Fields] AND "cas"[All Fields] AND "systems"[All Fields]) OR "crispr cas systems"[All Fields]) OR (("cells"[MeSH Terms] OR "cells"[All Fields] OR "cell"[All Fields]) AND "nucleus"[All Fields] AND ("genetic"[All Fields] OR "genetical"[All Fields] OR "genetically"[All Fields] OR "genetics"[MeSH Subheading] OR "genetics"[All Fields] OR "genetics"[MeSH Terms]) AND ("cells, cultured"[MeSH Terms] OR ("cells"[All Fields] AND "cultured"[All Fields]) OR "cultured cells"[All Fields] OR ("cells"[All Fields] AND "cultured"[All Fields]) OR "cells cultured"[All Fields])) OR (("gene expression"[MeSH Terms] OR ("gene"[All Fields] AND "expression"[All Fields]) OR "gene expression"[All Fields]) AND "profiling*"[All Fields] AND ("gene knockout techniques"[MeSH Terms] OR ("gene"[All Fields] AND "knockout"[All Fields] AND "techniques"[All Fields]) OR "gene knockout techniques"[All Fields])) OR ("rna seq"[MeSH Terms] OR "rna seq"[All Fields] OR ("rna"[All Fields] AND "seq"[All Fields]) OR "rna seq"[All Fields]) OR ("single cell analysis"[MeSH Terms] OR ("single cell"[All Fields] AND "analysis"[All Fields]) OR "single cell analysis"[All Fields] OR ("single"[All Fields] AND "cell"[All Fields] AND "analysis"[All Fields]) OR "single cell analysis"[All Fields]) OR ("genetic"[All Fields] OR "genetical"[All Fields] OR "genetically"[All Fields] OR "genetics"[MeSH Subheading] OR "genetics"[All Fields] OR "genetics"[MeSH Terms])) AND (("heart"[MeSH Terms] OR "heart"[All Fields] OR "hearts"[All Fields] OR "heart s"[All Fields]) AND "failure*"[All Fields])) OR ("heart ventricles"[MeSH Terms] OR ("heart"[All Fields] AND "ventricles"[All Fields]) OR "heart ventricles"[All Fields]) OR ("myocardium"[MeSH Terms] OR "myocardium"[All Fields] OR "myocardiums"[All Fields]) OR ("myofibroblastic"[All Fields] OR "myofibroblasts"[MeSH Terms] OR "myofibroblasts"[All Fields] OR "myofibroblast"[All Fields]) OR ("cardiomyocyte s"[All Fields] OR "cardiomyocytic"[All Fields] OR "myocytes, cardiac"[MeSH Terms] OR ("myocytes"[All Fields] AND "cardiac"[All Fields]) OR "cardiac myocytes"[All Fields] OR "cardiomyocyte"[All Fields] OR "cardiomyocytes"[All Fields]) OR ("cardiomyopathy, dilated"[MeSH Terms] OR ("cardiomyopathy"[All Fields] AND "dilated"[All Fields]) OR "dilated cardiomyopathy"[All Fields] OR ("dilated"[All Fields] AND "cardiomyopathy"[All Fields])) OR ("cardiovascular diseases"[MeSH Terms] OR ("cardiovascular"[All Fields] AND "diseases"[All Fields]) OR "cardiovascular diseases"[All Fields] OR ("cardiovascular"[All Fields] AND "disease"[All Fields]) OR "cardiovascular disease"[All Fields]) OR ("cardiomyopathy, dilated"[MeSH Terms] OR ("cardiomyopathy"[All Fields] AND "dilated"[All Fields]) OR "dilated cardiomyopathy"[All Fields] OR ("dilated"[All Fields] AND "cardiomyopathy"[All Fields])) OR ("muscular dystrophy, duchenne"[MeSH Terms] OR ("muscular"[All Fields] AND "dystrophy"[All Fields] AND "duchenne"[All Fields]) OR "duchenne muscular dystrophy"[All Fields] OR ("duchenne"[All Fields] AND "muscular"[All Fields] AND "dystrophy"[All Fields] AND "dmd"[All Fields]) OR "duchenne muscular dystrophy dmd"[All Fields]) OR ("dystrophin"[MeSH Terms] OR "dystrophin"[All Fields] OR "dystrophin s"[All Fields] OR "dystrophine"[All Fields] OR "dystrophins"[All Fields]) OR ("cardiomyopathie"[All Fields] OR "cardiomyopathies"[MeSH Terms] OR "cardiomyopathies"[All Fields] OR "cardiomyopathy"[All Fields]) OR ("muscular dystrophy, duchenne"[MeSH Terms] OR ("muscular"[All Fields] AND "dystrophy"[All Fields] AND "duchenne"[All Fields]) OR "duchenne muscular dystrophy"[All Fields] OR ("muscular"[All Fields] AND "dystrophy"[All Fields] AND "duchenne"[All Fields]) OR "muscular dystrophy duchenne"[All Fields]) OR (("cardiomyopathie"[All Fields] OR "cardiomyopathies"[MeSH Terms] OR "cardiomyopathies"[All Fields] OR "cardiomyopathy"[All Fields]) AND "dilated*"[All Fields])) AND ((y_5[Filter]) AND (clinicaltrial[Filter] OR randomizedcontrolledtrial[Filter])).

Google Scholar and Cochrane Central

Search keywords: CRISPR OR CRISPR/Cas9 OR Gene editing OR Phenotypic screening OR Drug Screening OR Gene knock-in OR Gene knock-out AND Cardiomyopathy OR Dilated OR LV failure OR LV dysfunction OR Duchenne’s OR Dystrophin OR Genetic cardiomyopathy.

Search results: 21,055 results.
